# No Association between Low Birth Weight and Cardiovascular Risk Factors in Early Adulthood: Evidence from São Paulo, Brazil

**DOI:** 10.1371/journal.pone.0066554

**Published:** 2013-06-17

**Authors:** Filumena Maria Gomes, S. V. Subramanian, Ana Maria de Ulhôa Escobar, Maria Helena Valente, Sandra Josefina Ferraz Ellero Grisi, Alexandra Brentani, Günther Fink

**Affiliations:** 1 Centro de Saúde Samuel Barnsley Pessoa, Faculdade de Medicina da Universidade de São Paulo, São Paulo, Brazil; 2 Department of Society, Human Development, and Health, Harvard School of Public Health, Boston, Massachusetts, United States of America; 3 Centro de Saúde Samuel Barnsley Pessoa, Faculdade de Medicina da Universidade de São Paulo, São Paulo, Brazil; 4 Department of Pediatrics, Faculdade de Medicina da Universidade de São Paulo, São Paulo, Brazil; 5 Department of Global Health and Population, Harvard School of Public Health, Boston, Massachusetts, United States of America; University of Miami, United States of America

## Abstract

**Background:**

A growing literature suggests that low birth weight increases the risk of poor health outcomes in adulthood. We tested this hypothesis among young adults living in São Paulo State, Brazil.

**Methods and Findings:**

To identify the effects of low birth weight on young adulthood outcomes, a medical assessment of 297 individuals born between 1977 and 1989 was conducted at a primary care unit in São Paulo State, Brazil. We analyzed body mass index (BMI), waist-hip ratio, blood pressure, fasting glucose and total cholesterol levels using linear and logistic regressions. Low birth was negatively associated with BMI (β = −2.0, 95% CI: −3.69, −0.27, p = 0.02), fasting glucose levels (β = −1.9, 95% CI: −3.9, −0.07, p = 0.05), waist-hip ratio (β = −0.03, 95% CI: −0.07, −0.01, p = 0.10), systolic blood pressure (β = −3.32, 95% CI: −7.60, 0.96, p = 0.12), and total cholesterol levels (β = −3.19, 95% CI: −16.43, 10.05, p = 0.636). Low birth weight was also associated with lower odds of young adults being overweight and obese, but neither association was statistically significant. Weight gain in the first 12 months of life was associated with higher adult BMI (β = 0.79, 95% CI: −0.0455, 1.623, p = 0.064) and blood pressure (β = 2.79, 95% CI: 0.22, 5.35, p = 0.034). No associations were found between low birth weight and early life (catch-up) growth.

**Conclusions:**

Low birth weight was not associated with poor health outcomes among young adults in Brazil. These results appear inconsistent with the original Barker hypothesis, but will need to be corroborated in larger samples with longer follow-ups to allow a more general evaluation of the validity of the hypothesis in low and middle income countries.

## Introduction

Following the Barker hypothesis, a large and growing literature has investigated the associations between low birth weight and adult health. According to the Barker hypothesis [1], intrauterine environment disorders as well as the subsequent compensatory growth lead to greater risk of obesity, insulin resistance, diabetes, hypertension and ischemic heart disease in adult life [1]–[4]. The most commonly used measure of intrauterine growth disorders is low birth weight. Low birth weight, generally defined as any birth below 2,500 grams, can occur in premature babies born before the 37th week of pregnancy, or in babies who are born in the expected time interval; typically this second group of children are presumed to have experienced intrauterine growth restrictions [5].

A large number of studies have postulated a negative theoretical link from low intrauterine growth restrictions to later life growth in the vein of the original work by Barker [1], [6]–[8]; most of the empirical studies are based on high income countries [9] and have resulted in highly varying results. Yu et al. [10] review the empirical associations between low birth weight and later life obesity, and find that the evidence is inconclusive in a majority of published studies.

Few studies have analyzed the relationship between low birth weight and cardiovascular risk factors beyond basic anthropometric measures in a developing country context. One of the best non-developed country studies is the Pelotas cohort study in Southern Brazil [11]–[13]. While the study suggests a generally positive relation between birth weight and weight in young adulthood, the study does not specifically focus on low birth weight, and provides limited information on other early markers of metabolic syndrome. The objective of this study was to corroborate the existing evidence through detailed medical exams among low and normal birth weight individuals. To do so, a detailed clinical checkup was conducted with 297 individuals born in São Paulo, Brazil between 2008 and 2010.

## Methods

### Ethics Statement

All participants signed a written consent form. The consent form model, as well as the study were approved by the University of São Paulo Medical School's internal review board, on 10/22/2008, process number 0785/08E.

### Study Context and Objectives

We analyzed data collected through the Centro de Saúde Escola Butantã (CSEB) located in the western region of São Paulo Municipality. The CSEB is one of fourteen primary care units in the western region, covering a population of approximately 40,000 individuals. The total population of the western region is about 377,000, which corresponds to about 4% of the population of São Paulo municipality, which is currently estimated at 11.3 Million. With a crude birth rate of 15.2 and an infant mortality rate of 17 per 1000 live births, the western region is very close to the national average of Brazil with respect to population health and age structure. Over the past decades, both the region and the country have made remarkable progress with respect to population health, with infant mortality dropping from 46 deaths per 1000 in 1990 to 17 deaths per 1000 in 2009 at the national level [14]. The objective of the study was to observe adult health outcome for children born at the CSEB.

### Procedures

The first records collected at the center date from 1977; the latest records considered for the study were children born in 1989. Complete birth records (paper files) could be located for 632 individuals registered with the CSEB. All 632 individuals were invited to participate in one of three assessment sessions; 297 (47%) took up the offer and were interviewed and medically assessed at the CSEB between 2008 and 2010. During the assessment, physical examinations were conducted to measure height, weight, blood pressure, heart rate, a standard battery of cholesterol measures (total cholesterol, low-density lipoproteins (LDL) cholesterol, high-density lipoproteins (HDL) cholesterol, and triglycerides) as well as standard blood glucose measures. Individuals also completed a short follow-up questionnaire collecting information regarding their educational level, wealth and income. Information on birth conditions as well as weight and height over the first two years of life was extracted from the patient’s medical records.

### Data Analysis

Following Yusuf et al. [15] we analyzed a range of risk factors associated with cardiovascular health problems: body-mass-index (BMI), waist-hip ratio (WHR), blood pressure, total cholesterol levels and fasting glucose levels. Individuals with a BMI of 25 or higher were classified as overweight; individuals with a BMI of 30 or higher were classified as obese. Following the approach of the INTERHEART study [15] we classified individuals as abdominally obese if their WHR was 0.90 or higher. Individuals were considered hypertensive if their systolic blood pressure was 130 mm Hg or higher. Total cholesterol levels were coded as high if they exceeded 200 mg/dl, and fasting glucose levels were coded as high if they exceeded 100 mg/dL as suggested by the American Diabetic Association (ADA).

Empirical analysis was divided into three parts. Given the relatively high attrition rates, we used weight and gender data available from the original birth records to investigate whether patterns of study participation. In the second part, descriptive statistics were computed by study group; last multivariate analysis was conducted using basic linear regression models. For the multivariate analysis, both continuous and binary outcome measures were considered. Separate models were estimated to investigate the effects of physical growth in the first 12 months of children’s life on adult health outcomes.

All analysis was conducted using the Stata © 11 statistical software package.

## Results

Table 1 shows the results of the attrition analysis. The dependent variable in the logistic regressions conducted was a binary indicator which equaled one if the subject came to the hospital for the clinical assessment. As Table 1 shows, study participation neither showed a systematic association with low birth weight, nor with the gender of the subject.

**Table 1:** Attrition Analysis.

Dependent:	Study Participation		
	(1)	(2)	(3)
Low birth weight	0.927		0.933
	(0.571–1.505)	(0.572–1.521)	
Female		1.162	1.163
		(0.825–1.637)	(0.825–1.638)
	0.895	0.792	0.799
	(0.757–1.057)	(0.595–1.055)	(0.597–1.069)
			
Observations	632	628	628

*Notes*: 95% confidence intervals in parentheses.

Table 2 shows descriptive statistics of core sample of 297 subjects who underwent the clinical assessment at the hospital. Out of the 297 individuals assessed, 33 (11%) had a birth weight of less than 2500 grams. 26.7% of children were delivered after gestation period shorter than 37 weeks, and a, at least for the period, remarkable 30% of children were delivered by Caesarean section. Out of the 33 births classified as low birth weight births, only 6 births were delivered prematurely (gestation period <37 weeks), suggesting intrauterine growth restrictions as principal driver of low birth weight.

**Table 2:** Main Sample Characteristics.

	Normal birth weight *N = 264*		Low birth weight *N = 33*	
**Birth characteristics**				
Gestation <37 weeks	66	(27.8)	6	(18.2)
Cesarean	82	(31.1)	7	(21.2)
**Sociodemographic characteristics**				
Age in years, *mean (SD)*	26.0	(2.9)	26.5	(2.5)
Female	190	(72.0)	21	(63.6)
Education below 11 years, *n (%)*	68	(25.8)	5	(15.2)
Education 11 years, *n (%)*	150	(56.8)	23	(69.7)
Education above 11 years, *n (%)*	46	(17.4)	5	(15.2)
Wage relative to minimum, *mean (SD)*	1.4	(1.1)	1.3	(1.3)
Radios	1.3	(0.8)	1.0	(0.6)
TVs	1.9	(0.9)	1.7	(0.8)
Cars	0.5	(0.7)	0.4	(0.7)
**Anthropometry, BMI and Waist-hip Ratio**				
Height in cm, *mean (SD*)	165.0	(7.9)	164.8	(12.1)
Weight in kgs, *mean (SD)*	68.9	(15.9)	63.5	(16.5)
BMI, *mean (SD)*	25.1	(5.4)	23.1	(4.1)
Waist-hip ratio	0.86	(0.12)	0.84	(0.11)
**Medical examination outcomes**				
Systolic blood pressure, mean (SD)	113.6	(12.7)	111.5	(12.2)
Diastolic blood pressure, mean (SD)	71.0	(9.5)	68.4	(9.3)
Fasting glucose, mean (SD)	83.1	(7.8)	80.9	(4.9)
Total cholesterol, mean (SD)	162.0	(32.0)	157.3	(33.7)

Given that only subjects registered with the health care unit were eligible for the study, a large majority (71%) of study participants was female. Most participants completed some secondary schooling (11 years), with only 17.2% of respondents completing higher education. The average BMI in the sample is close to 25, and the average wage-to-hip ratio is 0.85. About 11% of the population has total cholesterol levels above 200 mg/dL, and only 1% of the subjects display elevated glucose levels. Using the international BMI standards, 29.9% of respondents would be classified as overweight, while 13.5% of respondents were classified as obese.

Table 3 shows the main multivariate results. On average, individuals with low birth weight are less obese, and have lower blood pressure, fasting glucose and total cholesterol levels – these differences are statistically significant for BMI and fasting glucose at conventional levels. It is worth highlighting that relatively large effect sizes are needed here for statistically significance; with only 33 cases of low birth weight, the study is powered to detect 0.5 standard deviations with a power of 0.8 in unadjusted models. The estimated coefficients change only marginally when other control variables are included in the analysis.

**Table 3:** Low Birth Weight and Continuous Health Outcomes.

	Unadjusted Effect of low birth weight	Adjusted Effect of low birth weight^a)^
Dependent:	(1)	(2)
BMI	−2.030**	−1.979**
	(−3.575–−0.485)	(−3.685–−0.273)
Waist-hip ratio	−0.0260	−0.0346
	(−0.0649–0.0129)	(−0.0764–0.00734)
Systolic blood pressure	−2.048	−3.321
	(−6.449–2.352)	(−7.604–0.962)
Fasting glucose	−2.208**	−1.898*
	(−4.131–−0.286)	(−3.868–0.0724)
Total cholesterol	−4.693	−3.188
	(−16.75–7.368)	(−16.43–10.05)

Notes: a) Models are adjusted for gestation period, sex, educational attainment group, 5-year age group, individual wage, as well as household ownership of radios, TVs and cars.

*** p<0.01,

** p<0.05,

* p<0.1.

Table 4 shows the logistic regression model estimates of the association between low birth weight and the probability of individual health measures exceeding critical thresholds (binary outcome variables). While the overall results were directionally consistent with the linear regression results displayed in Table 2, none of the estimated odds ratios were statistically significant at conventional levels.

**Table 4:** Threshold Effects of Low Birth Weight.

	Unadjusted Effect of low birth weight	Adjusted Effect of low birth weight^a)^
Dependent:	(1)	(2)
BMI > = 25	0.581	0.595
	(0.266–1.271)	(0.261–1.354)
BMI > = 30	0.384	0.370
	(0.0879–1.674)	(0.0815–1.682)
Waist-hip ratio > = 0.9	0.799	0.686
	(0.331–1.931)	(0.271–1.738)
Systolic blood pressure > = 130	0.915	0.682
	(0.261–3.214)	(0.135–3.434)
Total cholesterol > = 200	1.163	1.193
	(0.380–3.557)	(0.334–4.256)

Notes: a) Models are adjusted for gestation period, sex, educational attainment group, 5-year age group, individual wage, as well as household ownership of radios, TVs and cars.

Reported coefficients are odds ratios. 95% confidence intervals in parentheses.

Table 5 summarizes the growth data collected for study participants during follow-up visits at the clinic over the first 3 years of life as well as the final adult height and weight. While children with low birth weight caught up with respect to height within the first 12 months of their life and displayed virtually no height differences as adults, an average weight differential of 5.5 kilograms (8%) persisted through early adulthood. With lower weight and equal height, low birth weight children displayed on average lower rates of overweight and obesity in early adulthood.

**Table 5:** Height and Weight Trajectories.

	Weight in kgs at				
	Birth	12 Months	24 Months	36 Months	Follow-up
Normal weight	3.2	9.5	12.0	14.2	68.9
Low birth weight	2.1	8.1	10.7	12.3	63.4
Relative weight	67.3%	85.8%	89.0%	86.8%	92.0%
	**Height in cm at**				
	**Birth**	**12 Months**	**24 Months**	**36 Months**	**Follow-up**
Normal weight	48.0	72.7	84.5	88.6	164.8
Low birth weight	45.4	70.5	81.2	86.3	163.3
Relative height	94.5%	97.0%	96.0%	97.4%	99.1%

**Figure 1:**
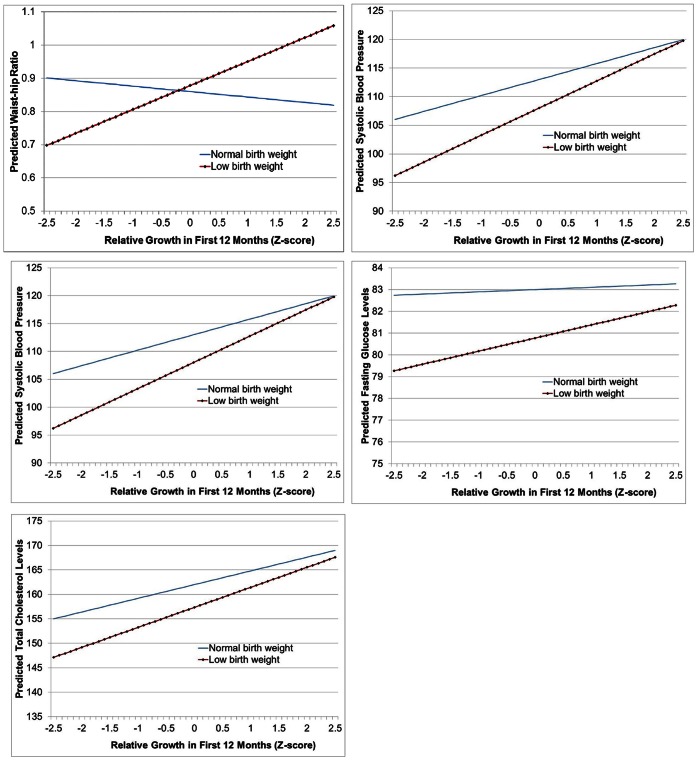
Predicted Associations between Early Life Growth and Health Outcomes by Birth Weight.

Table 6 shows the estimated associations between the continuous health outcomes and weight gain in the first 12 months of children’s life. Weight records were only available for children who visited the health center, for a total of 142 records. Column (1) of Table 6 shows the associations between low birth weight and the continuous health outcomes (similar to the ones shown in Table 3) conditional on weight gain in the first 12 months as well as an interaction term between weight gain and low-birth weight. The overall association between low birth weight and 12-months-growth was negative (β = −0.25, 95% CI: −0.77, 0.27, p = 0.341). With the smaller sample and additional controls, all associations lose statistical significance, even though the estimated magnitudes change only little. Growth in the first 12 months displays statistically significant associations with BMI (β = 0.79, 95% CI: −0.0455, 1.623, p = 0.064) and blood pressure (β = 2.79, 95% CI: 0.22, 5.35, p = 0.034), but no statistically significant associations with waist circumference, cholesterol and fasting glucose levels. All estimated effects appear to increase (positively interact) with low birth weight, but the interaction was statistically significant only for waist circumference (β = 0.089, 95% CI: 0.02, 0.16, p = 0.011). Figure 1 illustrates the interactions between low birth weight and early childhood growth. Even though low birth weight unambiguously increases the negative effects of rapid growth in the first 12 months of life, this effect is generally dominated by the overall protective effects of low birth weight, so that health outcomes for low birth weight children are superior to normal birth weight children for nearly all of the observed range of growth in the first 12 months.

**Table 6:** Low Birth Weight, Early Life Growth and Continuous Health Outcomes.

	Adjusted Effect of LowBirth Weight	Adjusted Effect of Growth inFirst 12 Months of Life^a)^	Adjusted Interaction Effect (Growth X Low Birth Weight)
**Dependent:**	(1)	(2)	(3)
BMI	−1.701	0.789*	0.785
	(−4.142–0.739)	(−0.0455–1.623)	(−0.883–2.452)
Waist-hip ratio	0.0181	−0.0164	0.0885**
	(−0.0644–0.101)	(−0.0392–0.00634)	(0.0203–0.157)
Systolic blood pressure	−4.996	2.786**	1.935
	(−12.17–2.179)	(0.215–5.357)	(−5.194–9.063)
Fasting glucose	−2.226	0.104	0.499
	(−5.478–1.025)	(−2.196–2.404)	(−2.429–3.428)
Total cholesterol	−4.649	2.801	1.289
	(−26.35–17.05)	(−3.719–9.320)	(−18.53–21.10)

Notes: a) Growth is measured in standard deviations relative to the age-group average. Models are adjusted for gestation period, sex, educational attainment group, 5-year age group, individual wage, as well as household ownership of radios, TVs and cars. Based on a reduced sample of 142 observations with complete early childhood growth data.

*** p<0.01,

** p<0.05,

* p<0.1.

## Discussion

The results presented in the previous section suggest that low birth weight is not associated with adverse cardiovascular risk profile during young adulthood, with indications that they may be even protective. The protective effects appear to be particularly strong for BMI. While this result is consistent with previous evidence based on the Pelotas study in Brazil [12], [13], it seems remarkable that intrauterine growth restrictions do not appear to show any association with metabolic syndrome despite the rather high prevalence of obesity and overweight among young adults.

One important difference between the cohort analyzed and a majority of existing studies including the Pelotas study [16] is that low birth weight in the sample analyzed is not associated with more rapid (“catch-up”) growth in infancy. Catch-up growth has been highlighted as major risk factor for adolescent obesity in previous research [17], and was also found to be associated with increased BMI and blood pressure in this study. The fact that catch-up growth was not associated with low birth weight in this study is likely to at least partially explain why the generally well established link between low birth weight and adult health outcomes was not found in this paper. In a recent analysis of 5 developing country cohorts, Victora et al. [18] find positive associations between low birth weight, blood pressure and glucose levels once BMI is controlled for. Similar patterns were not apparent in our data, and further research will be needed to better understand the causal mechanisms from early life growth restrictions to adult health in developing country settings.

While the presented study has only limited power to investigate interactions between intrauterine growth restrictions, the results found in this study suggest that a combination of intrauterine growth restrictions with fast early life growth (rather than growth restrictions alone) may be harmful for children’s physical development; this is consistent with a more recent generation of thrifty phenotype models outlined in Wells [19], which stress the important interactions between metabolic capacity and metabolic load in the early stages of development. At the same time, the estimated associations suggest that the net effect of low birth weight on health is positive for most of the observed range of child growth. Further and more adequately powered studies will be needed to better understand the complex interactions between intra-uterine and early life growth.

Apart from the small sample size, the study design has several important limitations. First, while detailed information on subjects’ education, income and living conditions was collected, no information on parental background is available. A large literature has documented the positive association between parental socioeconomic status and birth weight [20]. To the extent that this positive association holds in the sample analyzed, the positive association between birth weight and health outcomes may underestimate the true protective effect of low birth weight in this environment. On the other hand, one could argue that individuals from poor family backgrounds may receive less financial support from outside their core families or may be obliged to provide support to other members of the extended family, so that poor family background may have a negative impact on calorie intake.

This directly links to a second limitation of the study. While the lower levels of fasting glucose and total cholesterol among the low birth weight group could be interpreted as evidence of better nutrition in this subgroup, no data was collected on actual calorie intake. Therefore, the available data does not allow us to distinguish nutrition behavior from genetic or metabolic differences.

Third, given that participating in the study required visiting the health center on specific dates, only about half of the targeted subjects were able to participate in the study. While we did not find any systematic differences with respect to sex and birth weight, participating subjects may differ with respect to other dimensions not related to low birth weight; it is not clear, however, which variables would fall under this category, and how they would affect the estimated coefficients.

The small area covered by the sample clearly also raises the question of how representative the results presented are for the country or region. As discussed in the earlier sections of this paper, the area analyzed is representative of the larger São Paulo region, which accounts for about 15% of the national population, and for about 20% of the urban population – according to the latest estimates, 87% of the Brazilian population lives in urban areas. Relative to the other states, average incomes and educational attainment in São Paulo are undoubtedly higher [21]. Nevertheless, the findings reported are consistent with another study conducted in a very different setting in Brazil [13], which suggests that the results may hold beyond the specific setting analyzed.

Last, and most importantly, the evidence presented does not allow to directly measure the later life health differentials observed in Barker’s original work [1]. Even if low birth weight children display better health as young adults, a negative association between low birth weight and cardiovascular health problems may arise later in life and not be detectable by standard medical exams in early adulthood. If later life differences should occur, it will not be straightforward to attribute these differences to suboptimal early growth environments given that the overall trajectories of normal and low-birth weight cohorts are likely display substantial non-random variations over time.

Despite these important limitations, the results presented in the paper represent an important contribution to the literature testing the validity of the Barker hypothesis in low and middle income country settings. The lacking association between low birth weight and adverse early adulthood outcomes found in this paper corroborates previous findings from similar settings [11]–[13], and further highlights the need for larger and more long-term studies in this area.

## References

[1] Barker D, Winter P, Osmond C, Margetts B, Simmonds S (1989) Weight in infancy and death from ischaemic heart disease. Lancet 2: 577–580. 2570282 10.1016/s0140-6736(89)90710-1

[2] Barker D, Eriksson J, Forsén T, Osmond C (2002) Fetal origins of adult disease: strength of effects and biological basis. International Journal of Epidemiology 31: 1235–1239. 12540728 10.1093/ije/31.6.1235

[3] Barker DJP (1992) The fetal and infant origins of adult disease. London: BMJ Books. 10.1136/bmj.301.6761.1111 PMC1664286 2252919

[4] Barker DJP (1997) Maternal Nutrition, Fetal Nutrition, and Disease in Later Life. Nutrition Abstracts and Reviews 13. 10.1016/s0899-9007(97)00193-7 9290095

[5] Stevens LM, Lynm C, Glass RM (2002) Low Birth Weight. JAMA 287.

[6] Xita N, Tsatsoulis A (2010) Fetal origins of the metabolic syndrome. Annals of the New York Academy of Sciences 1205: 148–155. 20840267 10.1111/j.1749-6632.2010.05658.x

[7] Yajnik C, Deshmukh U (2008) Maternal nutrition, intrauterine programming and consequential risks in the offspring. Rev Endocr Metab Disord 9: 203–211. 18661241 10.1007/s11154-008-9087-z

[8] Johnson RC, Schoeni R (2011) The Influence of Early-Life Events on Human Capital, Health Status, and Labor Market Outcomes Over the Life Course. The BE Journal of Economic Analysis & Policy: Advances 11. 10.2202/1935-1682.2521 PMC3569741 23412970

[9] Baird J, Fisher D, Lucas P, Kleijnen J, Roberts H, et al (2005) Being big or growing fast: systematic review of size and growth in infancy and later obesity. BMJ (Clinical Research Ed) 331: 929. 10.1136/bmj.38586.411273.E0 PMC1261184 16227306

[10] Yu Z, Han S, Zhu G, Zhu C, Wang X, et al (2011) Birth weight and subsequent risk of obesity: a systematic review and meta-analysis. Obes Rev 12: 525–542. 21438992 10.1111/j.1467-789X.2011.00867.x

[11] Gigante DP, Minten GC, Horta BL, Barros FC, Victora CG (2008 ) Nutritional evaluation follow-up of the 1982 birth cohort, Pelotas, Southern Brazil. Rev Saude Publica (Suppl 2): 60–69. 10.1590/s0034-89102008000900009 PMC2671135 19142346

[12] Monteiro P, Victora C, Barros F, Monteiro L (2003) Birth size, early childhood growth, and adolescent obesity in a Brazilian birth cohort. Int J Obes Relat Metab Disord 27: 1274–1282. 14513077 10.1038/sj.ijo.0802409

[13] Victora C, Barros F (2006) Cohort profile: the 1982 Pelotas (Brazil) birth cohort study. International Journal of Epidemiology 35: 237–242. 16373375 10.1093/ije/dyi290

[14] Unicef (2012) Unicef Country Statistics.

[15] Yusuf S, Hawken S, Ôunpuu S, Dans T, Avezum A, et al (2004) Effect of potentially modifiable risk factors associated with myocardial infarction in 52 countries (the INTERHEART study): case-control study. The Lancet 364: 937–952. 10.1016/S0140-6736(04)17018-9 15364185

[16] Wells JCK, Hallal PC, Reichert FF, Dumith SC, Menezes AM, et al (2011) Associations of Birth Order With Early Growth and Adolescent Height, Body Composition, and Blood Pressure: Prospective Birth Cohort From Brazil. American Journal of Epidemiology 174: 1028–1035. 21940799 10.1093/aje/kwr232 PMC3658103

[17] Ong KKL, Ahmed ML, Emmett PM, Preece MA, Dunger DB (2000) Association between postnatal catch-up growth and obesity in childhood: prospective cohort study. BMJ (Clinical Research Ed) 320: 967–971. 10.1136/bmj.320.7240.967 PMC27335 10753147

[18] Victora CG, Adair L, Fall C, Hallal PC, Martorell R, et al (2008) Maternal and child undernutrition: consequences for adult health and human capital. The Lancet 371: 340–357. 10.1016/S0140-6736(07)61692-4 PMC2258311 18206223

[19] Wells JC (2011) The thrifty phenotype: An adaptation in growth or metabolism? Am J Hum Biol 23: 65–75. 21082685 10.1002/ajhb.21100

[20] Kramer MS (1987) Determinants of low birth weight: methodological assessment and meta-analysis. Bulletin of the World Health Organization 65: 663–737. 3322602 PMC2491072

[21] Binelliy C, Meghirz C, Menezes-Filhox N (2009) Education and Wages in Brazil. Mimeo.

